# The antidepressant effects of GM-CSF are mediated by the reduction of TLR4/NF-ĸB-induced IDO expression

**DOI:** 10.1186/s12974-019-1509-1

**Published:** 2019-06-01

**Authors:** Sara Hemmati, Mohammad Amin Sadeghi, Razieh Mohammad Jafari, Hasan Yousefi-Manesh, Ahmad Reza Dehpour

**Affiliations:** 1 Experimental Medicine Research Center, Tehran University of Medical Sciences, Tehran, Iran; 2 School of Medicine, Tehran University of Medical Sciences, Tehran, Iran; 3 Students’ Scientific Research Center, Tehran University of Medical Sciences, Tehran, Iran; 4 Department of Pharmacology, School of Medicine, Tehran University of Medical Sciences, Tehran, Iran

**Keywords:** Depression, Lipopolysaccharide, Granulocyte-macrophage stimulating factor, Indoleamine 2, 3-dioxygenase, NF-ĸB, Inflammation-induced depression, Forced swim test, Hippocampus, Neuroinflammation

## Abstract

**Background:**

Indoleamine 2, 3-dioxygenase 1 (IDO) is responsible for the progression of the kynurenine pathway. This pathway has been implicated in the pathophysiology of inflammation-induced depression in which conventional antidepressants are not effective. It has been reported that granulocyte-macrophage stimulating factor (GM-CSF) could interfere with the induction of IDO in septic patients. We hypothesized that GM-CSF could exert antidepressant effects through IDO downregulation in a model for acute inflammation-induced depression.

**Methods:**

To produce the model, lipopolysaccharide (LPS) (0.83 mg/kg) was administered intraperitoneally to mice. It has been well documented that LPS mediates IDO overexpression through TLR4/NF-ĸB signaling. In the treatment group, mice received GM-CSF (30 μg/kg, i.p.) thirty minutes prior to LPS injection. A validated selective serotonin reuptake inhibitor, fluoxetine (30 mg/kg i.p.), was also administered to an experimental group 30 min prior to LPS. Depressive-like behaviors were evaluated based on the duration of immobility in the forced swim test. To confirm that GM-CSF interferes with IDO induction in LPS treated mice, real-time PCR was used to quantify IDO mRNA expression. Furthermore, in order to study whether GM-CSF inhibits the TLR4/NF-ĸB signaling pathway, we measured levels ofpNF-ĸB and TLR4 by western blotting.

**Results:**

GM-CSF demonstrated significant antidepressant activity in the presence of LPS on immobility (*p* < .001) and latency (*p* = .010) times in the forced swim test. In contrast, fluoxetine did not show any antidepressant activity on either immobility (*p* = .918) or latency (*p* = .566) times. Furthermore, GM-CSF inhibited the increase in IDO mRNA (*p* = .032) and protein (*p* = .016) expression as a result of LPS administration. A similar trend was observed for TLR4 (*p* = .042) and pNF-ĸB (*p* = .026) expression as both proteins showed reduced expression levels in the GM-CSF-pretreated group compared to the untreated (LPS) group.

**Conclusion:**

Our results propose a promising antidepressant effect for GM-CSF possibly through the downregulation of IDO expression. This remedying effect of GM-CSF could be attributed to decreased amounts of TLR4 and active NF-ĸB in the treated mice.

## Introduction

Inflammation-induced depression is a debilitating psychiatric disorder which is caused by neurodegenerative metabolites in the CNS [1]. This type of depression is refractory to conventional medications such as selective serotonin reuptake inhibitors (SSRIs) because such treatments fail to affect the main trigger of the disease [2]. One of the most important pathways affected by the CNS inflammatory conditions is tryptophan metabolism [3]. Some catabolic metabolites of tryptophan in the kynurenine pathway are considered neurotoxic. One such metabolite, quinolinic acid, is the agonist of the N-methyl-D-aspartate (NMDA) receptor that leads to increased levels of reactive oxygen species (ROS) [4]. This oxidative stress may result in neuronal degeneration in the hippocampus and exacerbate depressive behaviors [5]. Aside from direct neurotoxic effects of ROS, they can induce well-known factors involved in depression such as the mitogen-activated protein kinase (ERK1/2) [6]. Inflammatory cytokines such as IFN-γ and TNF-α have been shown to enhance this pathway in cultured microglia by potentiating the expression of the pathway’s main controller enzyme, indoleamine 2,3-dioxygenase (IDO), through STAT1 activation [7]. In a similar manner, lipopolysaccharides (LPS) upregulate IDO levels through activating the TLR4/NF-ĸB signaling pathway [8]. A major site for IDO activation following LPS administration is the hippocampus [9, 10]. Importantly, the hippocampus is one of the most vulnerable areas of the brain to quinolinic acid [11] while simultaneously being the site for the highest production of neurotoxic metabolites of the kynurenine pathway following LPS challenge [12]. As a major site for major depressive disorder, inflammation and IDO activation in the hippocampus have been associated with depressive-like behaviors [13, 14]. Due to its mechanism of action, LPS is commonly used to induce acute inflammation-induced depressive-like behaviors in animal models in order to introduce novel therapeutic drugs [9].

Granulocyte-macrophage stimulating factor (GM-CSF) is well known for its effects on myeloid progenitor cells. Effects of GM-CSF are not confined to the bone marrow as it is reported to be a neuroprotective factor decreasing dementia [15]. In addition, it has demonstrated promising effects on increasing neuronal plasticity in animal stroke models [16]. These neuroprotective activities have been shown to result from decreased microglial activation, increased regulatory T cell activity, and increased brain-derived neurotrophic factor (BDNF) production [17, 18]. The GM-CSF receptor is composed of a specific α-chain (GM-CSFRα) and a β-chain which is also present in IL-3 and IL-5 receptors. GM-CSFRα expression is observed in various regions of the murine brain including the cortex and hippocampus [19]. Several signaling pathways have been reported to be activated by GM-CSF both in neurons and glial cells, including the JAK/STAT, MAP kinase, Akt/mTOR, and Bcl-2- and Bcl-xL-activating pathways [20–25].

In accordance with our study, it has been reported that GM-CSF treatment can reduce IDO activity and tryptophan catabolism in patients with septic shock [26]. We hypothesize that GM-CSF treatment can ameliorate depressive-like behaviors in the LPS-induced model of acute inflammation-induced depression in mice. To investigate whether the tryptophan catabolism pathway mediated the observed effects, IDO mRNA expression levels were assessed using real-time PCR. Furthermore, NF-ĸB activation, as determined by the phosphorylation of the p65 subunit, and TLR4 expression proteins were studied to investigate the signaling pathway involved.

## Methods

### Animals

We used adult male NMRI mice (Department of Pharmacology, Tehran University of Medical Sciences), weighing 20–25g. All experimental animals were maintained under standard housing conditions of temperature (21–23 °C), humidity (55%), and light/dark cycle (12-h light/dark), and had access to water and food ad libitum. All experiments were performed in accordance with the standards of animal care determined by the Council of Laboratory Animals of the Experimental Medicine Research Center, Tehran University of Medical Sciences, Tehran, Iran.

### Drugs and experimental groups

Our study consisted of 6 experimental groups. In the first group (LPS), mice were only injected with LPS (0.83 mg/Kg, i.p.) from Escherichia Coli O55:B5 (Sigma, St. Louis, MO, USA) in order to induce depressive behaviors. This dose of LPS had been determined to result in depressive-like behaviors in mice after 24 h by previous studies [9]. To demonstrate the unresponsiveness of this depression model to conventional SSRIs, the second group (LPS + Flx) received fluoxetine (30 mg/kg, i.p.) 30 min prior to LPS injection. In our third group (LPS + GM-CSF), GM-CSF (Peprotech, USA) was administered (30 μg/kg, i.p.) 30 min before LPS injection. A dose of 30 μg/kg has been shown to pass through the blood–brain barrier and exert neurotrophic and was confirmed to be effective in our dose-response studies (data not shown) [21, 27, 28]. Two other groups received GM-CSF (GM-CSF) or fluoxetine (Flx) followed by normal saline instead of LPS. All drugs were dissolved in normal saline (0.9%). The control group (control) was only injected with normal saline at 5 ml/kg (i.p).

### Behavioral assays

Depressive-like behaviors were measured using the forced swim test (FST) for mice 24 h after LPS administration. Increased immobility times in this test are considered to be indicative of depression [29]. Animals undergoing forced swimming were placed in a 25-cm-high open cylinder containing 19 cm of tap water at 25 °C. The immobility time of each mouse was calculated during the last 4 min of a 6-min test recording, allowing for an initial 2-min pretest period [30]. Latency times were measured from the beginning of this 4-min period. No climbing or diving activity was observed throughout the tests. Therefore, swimming times, which are complementary to immobility times, are not presented in the figures.

**Fig. 1:**
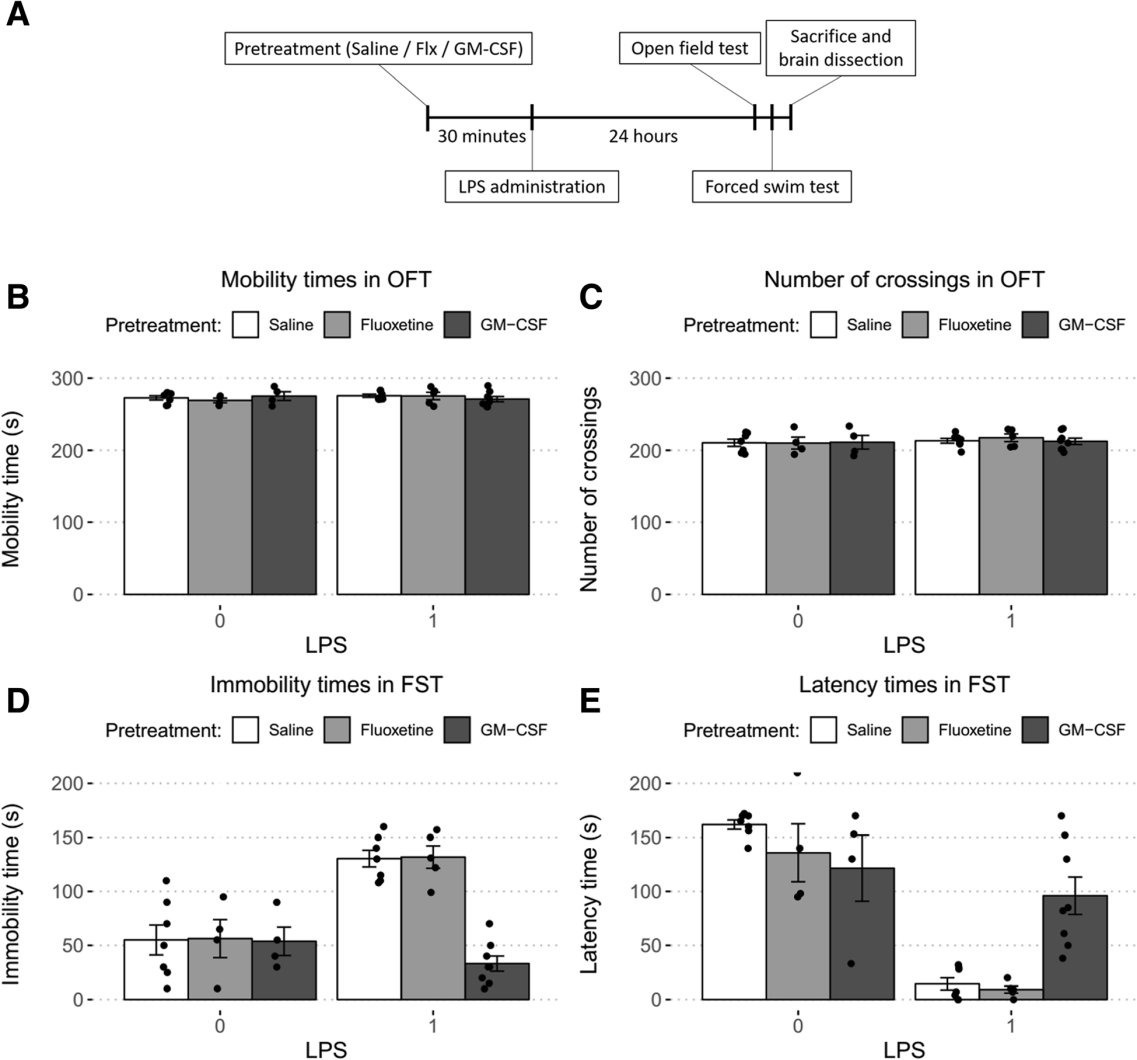
Comparison of the forced swim test results between study groups. For LPS treatment, 1 indicates administration while 0 indicates no administration. When combined with other treatments, LPS (0.83 mg/kg, i.p.) was administered 30 min after either GM-CSF (30 μg kg, i.p.) or fluoxetine (30 mg/kg, i.p.). Statistical significance was analyzed using two-way ANOVA followed by pairwise comparisons with the Tukey post hoc test. **a** The timeline of our experimental process. **b** Changes in mobility times in the OFT were not significant following fluoxetine (*p* = .499) or GM-CSF (*p* = .660) treatment regardless of LPS administration. **c** Changes in the number of gridline crossings were not significant following fluoxetine (*p* = .957) or GM-CSF (*p* = .929) treatment regardless of LPS administration. **d** Changes in immobility times in the FST were not significant following fluoxetine treatment regardless of LPS administration (*p* = .918). In contrast, GM-CSF treatment showed a significant interaction with LPS administration (*p* < .001). GM-CSF decreased immobility times significantly when followed by LPS administration (*p* < .001) but showed no effect when used alone (*p* = .940). **e** Latency times were measured from the beginning of the 4-min test period. Changes in latency times were not significant following fluoxetine treatment regardless of LPS administration (*p* = .322). However, GM-CSF treatment showed a significant interaction with LPS administration (*p* < .001). GM-CSF decreased latency times significantly when followed by LPS administration (*p* < .001) but showed no effect when used alone (*p* = .096)

The open field test was used to ensure that alterations in FST immobility duration were not related to changes in locomotor function. For this test, the floor of an acrylic plastic box (50 × 50 × 30 cm) was divided into 12 equal squares. The animals were gently placed in one of the corner squares, and locomotor activity was measured by counting the number of gridline crossings and duration of mobility during a 5-min period. All assessments were performed by a well-trained observer blind to the experiment. The mice in all experimental groups were determined to have normal locomotor ability just before the FST (Fig. 1b, c).

### Real-time PCR

Immediately after, the FST test, animals were decapitated and their hippocampi were rapidly dissected on an ice-cold surface and were flash-frozen in liquid nitrogen. After tissue homogenization, total cellular RNA was extracted in Trizol reagent. One microgram of total mRNA was reverse transcribed using cDNA kits. Specific mRNAs were amplified using the following primers: GAPDH as the housekeeping gene (Forward: TCAGAGCAAGAGAGGCATCC; Reverse: GGTCATCTTCTCACGGTTGG) and IDO (Forward: CATCAAGACCCGAAAGCAC; Reverse: CACGAAGTCACGCATCCTCT). Quantitative real-time PCR was performed using the Rotorgene 3000 thermocycler. Cycling conditions were consistent with previous studies [31]. Samples were run in triplicate and the 2^−ΔΔCt^ method was used to assess the mRNA expression fold-change in comparison to the control group.

### Western blotting

Mice hippocampi were homogenized in lysis buffer consisting of TRIS-HCl, SDS, DTT, glycerol, and NP40. The homogenates were then centrifuged at 15,000×*g* for 10 min at 4 °C, and the supernatants were used for SDS-PAGE. Ten micrograms of protein was resolved on 10% SDS-PAGE gel and moved onto polyvinylidene difluoride (PVDF) (Millipore (Germany)) membranes. Membranes were blocked for 120 min with 5% non-fat skimmed milk and incubated with the following primary antibodies overnight: TLR4, pNF-κB (p65), NF-κB, IDO1, GAPDH, and β-actin. All antibodies were purchased from Santa Cruz Biotechnology (Santa Cruz, CA, USA). Membranes were then washed 3 times with TBST (TBS+ tween 80) and incubated for 1 h at room temperature with secondary antibodies. Bands were visualized using the BM Chemiluminescence Western Blotting Kit acquired from Roche Diagnostics GmbH (Mannheim, Germany) and were detected using a gel documentation system. An open-source image-processing program, ImageJ, was used to quantify the optical densities of each band. The relative expressions of TLR4 and pNF-κB/total NF-κB were calculated and compared to the β-actin (TLR4 and pNF-κB/total NF-κB) or GAPDH (IDO1) as well as the control group.

### Statistics

The effects of treatment combinations on motor function in the open field and depressive-like behaviors forced swim tests were analyzed using two-way ANOVA. Due to the unbalanced number of subjects in each group, a type-III test was used for the combination of LPS and GM-CSF following the observation of a significant interaction between the two treatments while a type-II test was used for the combination of LPS and Flx due to the lack of a significant interaction [32]. The underlying assumptions of two-way ANOVA were validated for all data using the Levene and Shapiro-Wilk tests except for latency times in the experiment studying the combination of LPS and Flx. In this experiment, the residuals of the data were found not to be normally distributed. However, this may cause an only slight increase in type I error whereas our test results were negative. In addition, the overall robustness of two-way ANOVA against violations of normality [33] led us to not use a non-parametric test in this case. The results from the open field test were also analyzed using two-way ANOVA. IDO mRNA expression levels, and pNF-κB/total NF-κB and TLR4 expression were compared using one-way ANOVA followed by Tukey’s post hoc test for pairwise comparisons between groups. The underlying assumptions of ANOVA were upheld in these comparisons. All statistical analyses were performed using R, version 3.5.1.

## Results

### GM-CSF abrogates depressive-like behaviors in the forced swim test

Motor function, immobility, and latency times were compared between study groups of 4–8 mice. In the OFT, changes in mobility times and number of gridline crossings were not significant following fluoxetine (*p* = .499, *p* = .957, respectively) or GM-CSF (*p* = .660, *p* = .929, respectively) pretreatment regardless of LPS administration as determined by two-way ANOVA (Fig. 1b, c).

In the FST, a two-way ANOVA was conducted to analyze the effects of LPS and GM-CSF on depressive-like behaviors. There was a statistically significant interaction between the effects of GM-CSF and LPS on immobility time (*F* (1, 22) = 19.98, *p* < .001) and latency time (*F* (1, 22) = 16.44, *p* < .001). Simple main effects analysis showed that GM-CSF decreased immobility (*p* < .001) and latency times (*p* < .001) significantly when administered prior to LPS while showing no effect when not followed by LPS administration (*p* = .940 and *p* = .096, respectively) (Fig. 1d, e). In contrast, no interaction between LPS and Flx were observed in either FST immobility time (*F* (1, 19) = .00, *p* = .996) or FST latency time (*F* (1, 19) = 1.03, *p* = .322). Furthermore, main effects analysis showed significant effects for LPS on both immobility time (F (1, 19) = 37.73, *p* < .001) and latency time (*F* (1, 19) = 193.60, *p* < .001) while Flx pretreatment demonstrated no significant effect on either immobility (*F* (1, 19) = .01, *p* = .918) or latency (*F* (1, 19) = 2.13, *p* = .161) (Fig. 1d, e). These data indicate that GM-CSF treatment almost completely nullifies the effects of LPS administration on depressive-like behaviors.

### IDO expression is downregulated in GM-CSF-treated mice hippocampi

**Fig. 2:**
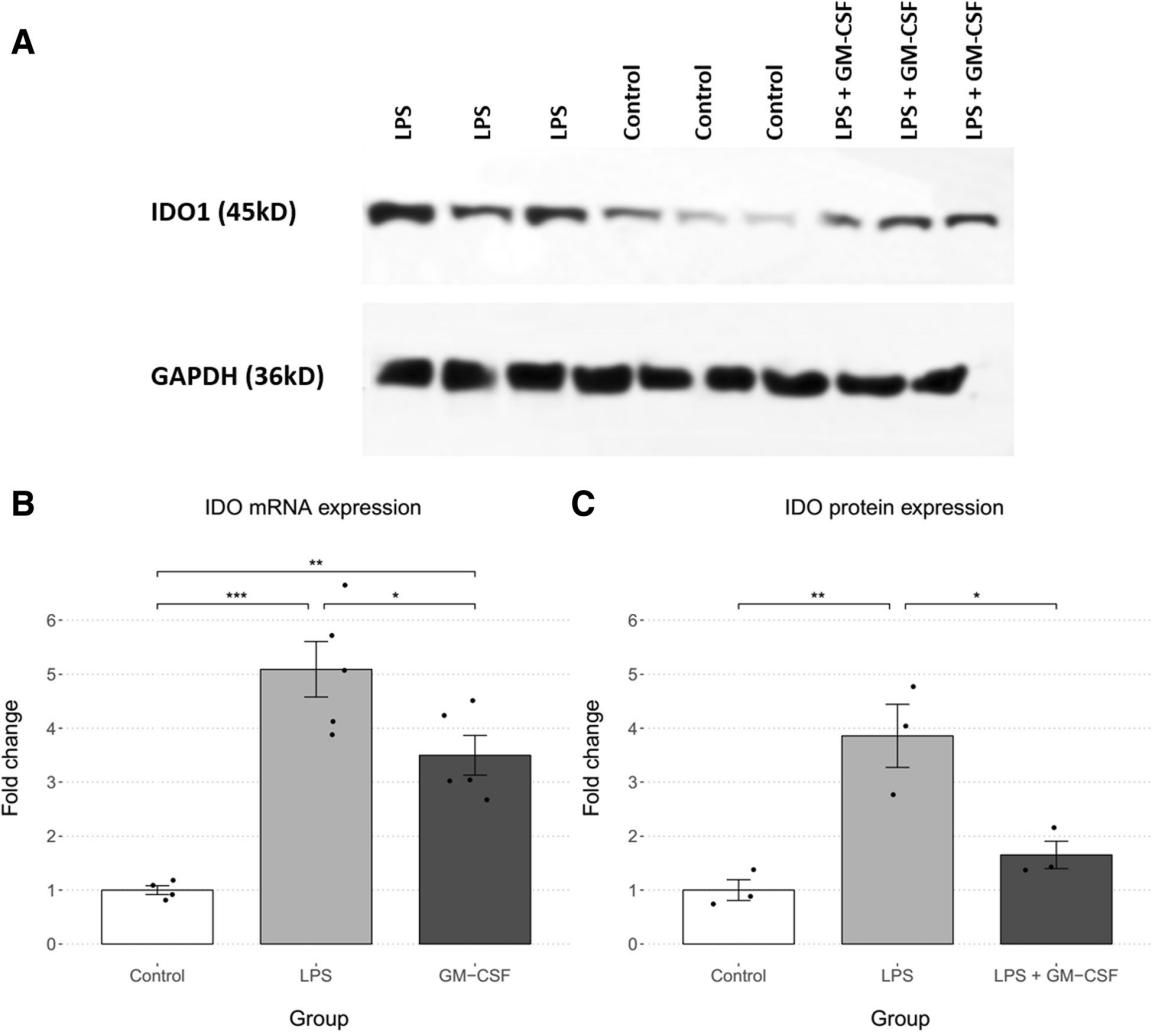
Comparison of IDO mRNA and protein expression between study groups. The LPS + GM-CSF group were pretreated with GM-CSF (30 μg kg, i.p.) 30 min prior to LPS injection (0.83 mg/kg, i.p.). Statistical significance was analyzed using one-way ANOVA followed by pairwise comparisons with the Tukey post hoc test and is depicted in both graphs as *p < .05, **p < .01, and ***p < .001. **a** The bands observed for each individual mouse in the western blot analysis of IDO. **b** IDO mRNA expression was significantly different between the three groups (*p* < .001). All pairwise comparisons were significant (control/LPS: *p* < .001; control/GM-CSF: *p* = .003; LPS/GM-CSF: *p* = .032). **c** IDO protein expression was significantly different between the three groups (*p* < .001). LPS significantly increased IDO expression (*p* = .005) while GM-CSF pretreatment inhibited this increase significantly (*p* = .016)

To assess the activity of the kynurenine pathway, we measured IDO mRNA levels and protein levels in mice hippocampi. Each study group consisted of 3–5 mice. As shown in Fig. 2b, expression of IDO mRNA significantly differed between the three groups as determined by one-way ANOVA (*F*(2,11) = 25.56, *p* < .001). All pairwise comparisons were significantly based on the Tukey post hoc analysis (control/LPS: 4.09 ± 1.55, *p* < .001; control/GM-CSF: 2.50 ± 1.55, *p* = .003; LPS/GM-CSF: 1.59 ± 1.46, *p* = .032).

IDO protein levels were also significantly different between the study groups (*F* (2, 6) = 15.09, *p* < .01). A Tukey post hoc analysis demonstrated a significant difference between the control and LPS groups (2.86 ± 1.67, *p* = .005) and between the LPS and LPS + GM-CSF groups (− 2.20 ± 1.67, *p* = .016). The difference between the control and GM-CSF groups was not significant (.65 ± 1.67, *p* = .497) (Fig. 2c). Therefore, GM-CSF decreases LPS-induced IDO mRNA and protein expression.

### Antidepressant effect of GM-CSF is potentially mediated by inhibiting LPS-induced activation of the TLR4/NF-κB signaling pathway

**Fig. 3:**
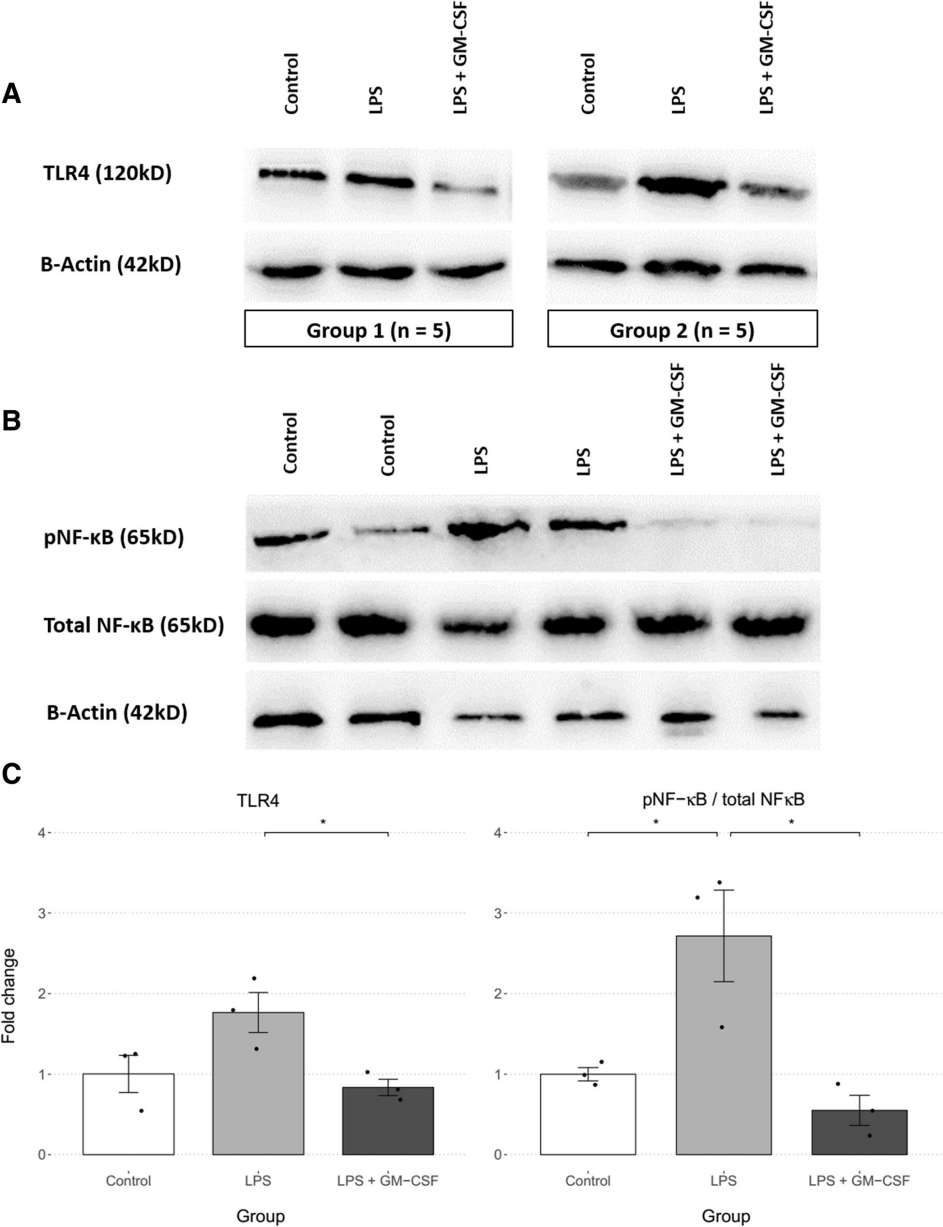
Comparison of TLR4 expression and NF-κB activation between study groups. The LPS + GM-CSF group were pretreated with GM-CSF (30 μg kg, i.p.) 30 min prior to LPS injection (0.83 mg/kg, i.p.). Statistical significance was analyzed using one-way ANOVA followed by pairwise comparisons with the Tukey post hoc test and is depicted in both graphs as *p < .05, **p < .01, and ***p < .001. **a** The TLR4 and β-actin bands observed for two of the three groups. Each group consisted of the aggregate of 5 mice hippocampi. **b** NF-κB expression and activation in mice hippocampi. Each lane contains the extract of a single mouse hippocampus. **c** The differences between the three groups were significant for both proteins (TLR4: *p* = .039; pNF-κB/total NF-κB: *p* = .010). GM-CSF pretreatment decreased TLR4 (*p* = .042) and pNF-κB (*p* = .011) expression significantly compared to the groups only receiving LPS

The expression levels of TLR4, pNF-κB, and total NF-κB in LPS and LPS + GM-CSF-treated mice were compared to the control group. For the TLR4 study, each study group consisted of 15 mice which were aggregated into three samples for SDS-PAGE and immunoblotting (Fig. 3a). In the NF-κB study, each lane contained a single hippocampal extract (Fig. 3b). There was a statistically significant difference between groups as determined by one-way ANOVA for TLR4 (*F* (2, 6) = 5.83, *p* = .039) and pNF-κB/total NF-κB (*F* (2, 6) = 10.76, *p* = .010). A Tukey post hoc analysis revealed that expression levels of both TLR4 (− .93 ± .89, *p* = .042) and pNF-κB/total NF-κB (− 2.16 ± 1.51, *p* = .011) were significantly lower in GM-CSF-treated mice compared to the untreated (LPS) groups. Furthermore, there was no significant difference between the GM-CSF-treated and control groups for either TLR4 (− .17 ± .89, *p* = .835) or pNF-κB/total NF-κB (− .45 ± 1.51, *p* = .654) (Fig. 3c).

## Discussion

Based on our results, GM-CSF showed novel promising antidepressant effects in a validated mouse model of inflammatory depression. Improvements in mice depressive-like behaviors were demonstrated by the reduction of immobility times in the FST test. Furthermore, the mechanism underlying our treatments was investigated by assessing IDO mRNA and TLR4 expression and NF-κB activation. Our results were consistent with previous studies which had indicated that LPS induces IDO expression through an upregulation of active pNF-κB and TLR4 in the mice hippocampi [8]. However, the suppressant effects of GM-CSF on TLR4, NF-κB activation, and hence the kynurenine pathway are demonstrated for the first time.

Importantly, the effects observed here may not be attributed to endogenous GM-CSF secretion in response to LPS administration. LPS injection has been reported to increase GM-CSF serum levels during the first 10 h as an inflammatory response only at high doses (3 mg/kg). Following the initial elevation, GM-CSF levels gradually decrease to baseline levels at 24 h post-injection [34, 35]. Furthermore, the elevation of serum GM-CSF levels has not been detected at different time points after LPS administration at a similar dose to our study [36]. Therefore, it may be concluded that the results observed in our study are mainly due to the exogenous GM-CSF administration. Nevertheless, administration of GM-CSF antagonists or use of GM-CSF knock-out (KO) mice may have enhanced the interpretation of our results. However, GM-CSF antagonists are not commercially available peptides and they are usually synthesized by individual laboratories. Furthermore, the most important obstacle for administering these antagonists in our study is their inability to pass the blood–brain barrier as they are actually large GM-CSF neutralizing antibodies. Furthermore, as mentioned, endogenous levels of GM-CSF seem to not play an important role in the results observed in our study; therefore, reducing the need for KO mice to some extent. These factors, combined with limitations in access to KO mice, forced us to forgo the use of these elements. Studies on the interactions of GM-CSF, the TLR4/NF-κB signaling system, and inflammation in general have produced seemingly contradictory results. Previous studies have reported that the GM-CSF receptor (GMR) triggers NF-κB activation in TF1 cells which are completely dependent on GM-CSF for growth [37]. Moreover, In one study, GM-CSF has shown pro-inflammatory functions in LPS-treated microglial cells in culture [38]. In the context of autoimmune diseases, GM-CSF has shown differing immunomodulatory (in diabetes type I and Crohn’s disease) and immunostimulatory (in rheumatoid arthritis and multiple sclerosis) effects [39–42]. Interestingly, a previous study by Kosloski et al. had shown both pro-inflammatory and anti-inflammatory changes, including TLR4 upregulation, in the mRNA expression profiles of mice substantia nigra following GM-CSF treatment. It is clear, therefore, that the biological effects of GM-CSF on inflammation are highly dependent on the microenvironment surrounding its site of action. Our study focuses on tryptophan catabolism in the hippocampus as it is the principal site damaged in neurodegenerative depression. Furthermore, we focused on IDO activation and tryptophan catabolism as a direct mediator of neuronal injury and degeneration regardless of the inflammatory state.

Evidently, IDO activation may be achieved through various pathways. One such pathway in epithelial cells is activated in response to inflammatory cytokines such as IFNγ and TNFɑ through JAK/STAT1 [43]. However, in one study, IDO expression remained unchanged in an animal model of cerebral ischemia in IFNγ^−/−^ mice compared with the wild types [44]. Another reported pathway for induction of IDO expression is the P38/MAPK pathway in peripheral mononuclear cells treated with LPS [8]. In our study, pP38 was not detected in the cortical and hippocampal tissues of any experimental group (data not shown). One important IDO-activating pathway involves pro-inflammatory cytokines which are stimulated by LPS challenge [12]. In general, GM-CSF is considered to promote inflammation and the secretion of pro-inflammatory cytokines such as TNF-α and IL-1β in immune cells [45]. The production of IL-1β by immune cells is also boosted through synergism of GM-CSF and LPS [46]. Furthermore, GM-CSF has been identified to be a major driver of inflammation in a rat model for experimental autoimmune encephalomyelitis (EAE) [47]. On the other hand, no significant changes in IL-1β, IL-6, or TNF-α were identified in the study by Kosloski et al. [17]. As a result, it does not seem plausible that attenuation of IDO activity following GM-CSF administration would be due to a reduction of pro-inflammatory cytokines. Therefore, TLR4/NF-κB signaling appears to be the most promising route for IDO elevation in the hippocampus of mice undergoing systemic LPS administration and consequent tryptophan catabolite formations.

Consistent with previous studies, animals receiving fluoxetine treatment before LPS administration did not show any improvement in depressive-like behaviors [48]. Such resistance to SSRI-therapy is also seen in 10–30% of patients with depression [2]. Interestingly, antidepressant-resistant patients have been shown to have higher levels of systemic inflammation [49]. Since systemic inflammation may in turn induce neurodegeneration [50], it could be postulated that treatment-resistant depression is more than just a simple depletion of neurotransmitters. The major problem underlying the disease is the degeneration of hippocampal neurons which are responsible for mood regulation. Importantly, GM-CSF has shown a neuroprotective effect in addition to (or, as a result of) its anti-inflammatory properties in models of spinal cord injury [51, 52]. These effects have also been observed in animal models of Parkinson’s disease [17, 53]. This might indicate that the neuroprotective effects of GM-CSF are not solely through its inhibitory effects on IDO and neurotoxic metabolite formation. For example, GM-CSF may induce BDNF production which would lead to improved functional recovery and regeneration of neurons following a CNS injury [52]. These findings may be in line with our study as GM-CSF treatment was able to completely prevent the formation of depressive-like behaviors even though IDO levels still increased upon LPS administration compared to the control group. GM-CSF is currently administered as a chemotherapeutic drug for neutropenia under the generic name Sargoramostim or Leukine. In further studies, the clinical utility of administering GM-CSF for refractory depression may be considered [54].

## Conclusion

**Fig. 4:**
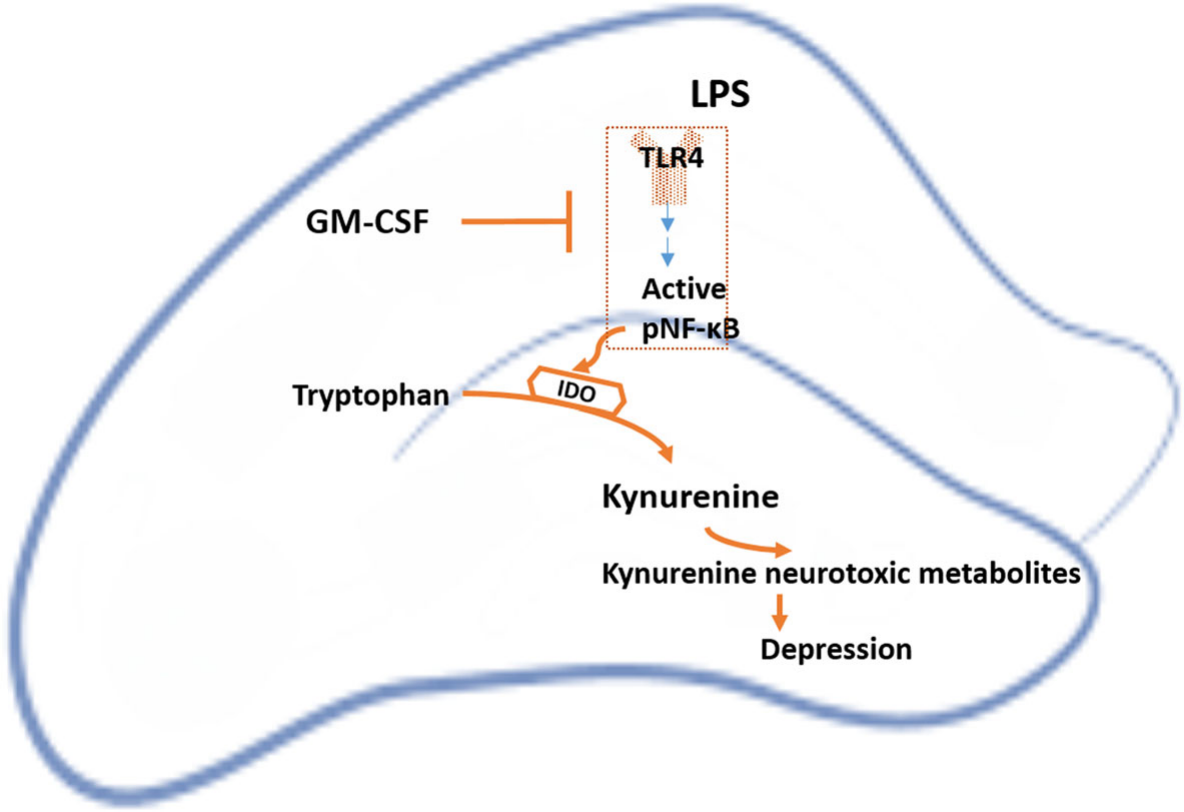
Schematic representation of the mechanism underlying the antidepressant effects of GM-CSF. The figure illustrates an overview of the kynurenine pathway which is involved in LPS-induced depression. Our findings indicated that GM-CSF interferes with this pathway as shown in the figure

In conclusion, we introduced GM-CSF as an optimal agent for treating depressive-like behaviors in an LPS-induced model of depression in mice. The mechanism underlying this model of acute inflammation-induced depression and our findings on GM-CSF interference with this pathway are depicted in Fig. 4.

## Data Availability

All the necessary data is included in the article. Further data will be shared by request.

## Abbreviations

FST: Forced swim test
GM-CSF: Granulocyte-macrophage stimulating factor
IDO: Indoleamine 2, 3-dioxygenase 1
LPS: Lipopolysaccharide
NMDA: N-methyl-D-aspartate
ROS: Reactive oxygen species
SSRIs: Selective serotonin reuptake inhibitors
